# Quadcopter UAVs Extended States/Disturbance Observer-Based Nonlinear Robust Backstepping Control

**DOI:** 10.3390/s22145082

**Published:** 2022-07-06

**Authors:** Ha Le Nhu Ngoc Thanh, Tuan Tu Huynh, Mai The Vu, Nguyen Xuan Mung, Nguyen Ngoc Phi, Sung Kyung Hong, Truong Nguyen Luan Vu

**Affiliations:** 1 Department of Mechatronics Engineering, Ho Chi Minh City University of Technology and Education (HCMUTE), Ho Chi Minh City 71307, Vietnam; thanh.hlnn@hcmute.edu.vn; 2 Faculty of Mechatronics and Electronics, Lac Hong University, Bien Hoa 810000, Vietnam; huynhtuantu@lhu.edu.vn; 3 School of Intelligent Mechatronics Engineering, Sejong University, Seoul 05006, Korea; maithevu90@sejong.ac.kr; 4 Faculty of Mechanical and Aerospace Engineering, Sejong University, Seoul 05006, Korea; xuanmung@sejong.ac.kr (N.X.M.); npnguyen@sejong.ac.kr (N.N.P.); 5 Department of Convergence Engineering for Intelligent Drone, Sejong University, Seoul 05006, Korea; 6 Faculty of Mechanical Engineering, Ho Chi Minh City University of Technology and Education (HCMUTE), Ho Chi Minh City 71307, Vietnam; vuluantn@hcmute.edu.vn

**Keywords:** trajectory tracking, extended state observer, disturbance observer, backstepping control, quadcopter UAV

## Abstract

A trajectory tracking control for quadcopter unmanned aerial vehicle (UAV) based on a nonlinear robust backstepping algorithm and extended state/disturbance observer (ESDO) is presented in this paper. To obtain robust attitude stabilization and superior performance of three-dimension position tracking control, the construction of the proposed algorithm can be separated into three parts. First, a mathematical model of UAV negatively influenced by exogenous disturbances is established. Following, an extended state/disturbance observer using a general second-order model is designed to approximate undesirable influences of perturbations on the UAVs dynamics. Finally, a nonlinear robust controller is constructed by an integration of the nominal backstepping technique with ESDO to enhance the performance of attitude and position control mode. Robust stability of the closed-loop disturbed system is obtained and guaranteed through the Lyapunov theorem without precise knowledge of the upper bound condition of perturbations. Lastly, a numerical simulation is carried out and compared with other previous controllers to demonstrate the great advantage and effectiveness of the proposed control method.

## 1. Introduction

The unmanned aerial vehicles also known as drones are a great achievement of science and technology. They are widely used in various fields of interest from academic research to industrial applications. Thus, there are an increasing number of scientific studies to expand the range of activities and enhance the operating capacity of these vehicles to reach their limit. The majority of this research has significantly been achieved in several major areas, such as a reliable controller construction [1], innovative airframe optimization design [2], efficient communication [3], and energy management for UAVs [4]. Nevertheless, there always exist many further challenges to construct an efficient flight controller to guarantee rigorous stability and rapid adaptation of the vehicle’s behavior in various flight environments due to different causes, such as: (i) The quadcopter UAV suffers from the intrinsic characteristic of an under-actuated system, meaning that the vehicle has less actuator control input than the number of degrees of freedom (DOF), which must be controlled. In addition, the various sources of parametric uncertainties and actuator failure are also considered as an essential characteristic needing to be handled; (ii) UAVs are also known as complex systems with an extremely nonlinear characteristic, open-loop instability, and strong coupled system with multiple input and multiple output (MIMO) variables in which the interaction between the rotational and translational dynamic model is highly complicated; (iii) The operational principle of multi-copter UAVs is based on the aerodynamic force generated by the spinning of multi-rotors and propellers. Thus, the vehicle is extremely sensitive to any interference of external disturbances, particularly gusts of wind.

To overcome the control challenge, a variety of works to improve the control performance of quadcopter UAVs have been conducted in recent decades. Many traditional linear control techniques have been introduced by using the Proportional Integral Derivative (PID) control law [5] and the Linear Quadratic Regulator (LQR) algorithm [6]. Although the PID and LQR can manage the hovering behavior of the vehicle and handle some simple actions, strict stability of the control system cannot be guaranteed in the presence of uncertainties and/or external perturbations. Furthermore, to design these linear controllers, a linearization process needs to be carried out surrounding single or multiple operating points. Thus, these methods still face the disadvantage of breaking down performance once the vehicle diverges from the considered points.

To attenuate the undesirable effects of nonlinearity parts and uncertainties/disturbances generated from various sources, plentiful control techniques have been introduced, such as a nonlinear control method presented in [7,8], whereby the controller guarantees a null tracking error and robust stability of the vehicle in the appearance of perturbations. In [9], an adaptive controller for trajectory tracking of UAVs is introduced by using an accurate parameterization algorithm. In [10], a nonlinear controller for the attitude and position tracking control of quadcopter UAVs is introduced by integration between a command-filtered backstepping algorithm and the gain scheduled method. In [11,12], a combination of model predictive algorithm and robust control ($H_{\infty }$) is conducted for trajectory tracking and attitude stabilization of a quadcopter UAV to obtain a null steady-state error under the effect of disturbances. However, in all these controller methods, the nominal control performance needs to be sacrificed to handle the stabilization of the control system in the presence of multiple uncertainties and/or disturbance sources.

With a simple procedure of construction and strict robustness to counteract uncertainties/disturbances, the sliding mode control (SMC) and extended SMC have been largely developed in the UAV control system, such as second-order sliding mode control [13,14,15,16], backstepping and integral SMC [17,18], and adaptive SMC [19,20]. It is well-known as a robust control in the SMC community, the nonsingular terminal SMC (NSTSMC) is an interesting technique [21], because the algorithm ensures both finite-time stability of sliding surface and the convergence of system states to desired trajectories. Motivated by the nonsingular terminal SMC and a nonlinear disturbance observer-based control [22,23], a composite control algorithm [24] is presented by an integration of the single hidden layer feedforward network and an NSTSMC to ensure the zero convergence of tracking error in a finite-time. In [25], another research based on a finite-time disturbance observer and NSTSMC is also developed to enhance the tracking control. However, these controller algorithms are only suitable for simple desired trajectories because several assumptions of these methods may not be practical in some cases. In [26], the concept of a virtual control variable is used to restructure the underactuated UAV system to obtain a fully-actuated model. Afterward, an advanced SMC is designed to enhance the control performance of the attitude and position subsystem. However, to reduce an inherent chattering effect in the SMC technique, the high gain parameters of the switching law must be avoided. Thus, this controller cannot strictly guarantee both precise tracking control and a strong anti-chattering effect.

The backstepping control technique and its extended methods are known as efficient recursive nonlinear control algorithms for a particular class of engineering system in which the controller is constructed from plentiful subsystem models. The process of designing a backstepping algorithm is to start from the origin of a complex system based on the form of state feedback, and substitute to the next step of the procedure. In this process, auxiliary variables and virtual control inputs are proposed to achieve the stable performance of each subsystem gradually. The process of backing out and substituting is terminated once an ultimate law is obtained [27,28,29]. Due to the advantage of the recursive structure, the backstepping method is renowned for its efficiency in the group of robust control techniques for complex engineering models, especially for unmanned aerial vehicles. Although there are many previous works that have solved the tracking control problem in both attitude stabilization and position control [30,31,32], the control performance is also ruined in the various working situations because it is not easy to estimate the variation in the uncertainties, exogenous perturbations, and actuator faults. To deal with this problem, the other extended backstepping controllers using disturbance observer and fault-estimation are employed [33,34,35,36]. In [37], an extended state observer is designed for online approximation in both the unknown velocity states and the perturbations affecting subsystem dynamics. Following, the dynamic surface controller is presented by using the estimated values to ensure the convergence of the tracking error to zero. However, in this research, the disturbance observer is constructed by using UAV position states computed by an integration of the velocity feedback. Obviously, these position states are not accurate in long-term operation because the accumulated error is always progressive during the integration process. Furthermore, the upper bound knowledge of uncertainties and/or exogenous perturbations is required for the observer design step. In [38], an active disturbance rejection control (ADRC) is introduced by using an auxiliary variable and cascade control method to ameliorate the robustness and anti-perturbation of the UAV control system. However, the drawback of ADRC is to cover some indeterminate coefficients consisting of tracking differentiators. Furthermore, the conflict between these parameters may occur in the controller tuning process.

The inspiration of this study is to deal with the aforementioned drawbacks of the existing research. A nonlinear robust backstepping control based on an extended state/disturbance observer (RBCESDO) for attitude stabilization and trajectory tracking control is presented to improve the performance of quadcopter UAVs under the various influences of uncertainties and/or external disturbances. In this method, the auxiliary variables, virtual controls, and ultimate control law are recursively constructed by using the approximated values of the system states and disturbances given by the ESDO. The contribution of this study can be stated as follows:

(i) The proposed algorithm overcomes the drawbacks of previous methods in the requirement of full state measurement. The ESDO is able to estimate the velocity state of the vehicle once this parameter cannot be directly measured. Thus, the implementation cost for data acquisition may be reduced and the influence of high measurement noise generated from the velocity sensor is also alleviated.
(ii) The unmeasured velocity states and lumped perturbations are estimated by the presented ESDO integrating with advantages of the recursive structure of the backstepping technique, the convergence of tracking errors is always guaranteed.
(iii) The numerical simulation is fully executed in both attitude and position control mode; these performance results are compared with other existing control methods to confirm the strict stability and efficiency of the presented control scheme in both the convergence error and anti-disturbance capacity.
(iv) Finally, unlike the existing methods, the upper bounds of the uncertainties and/or external disturbances are not demanded during the steps of designing the proposed control scheme.

The remainder of this article is organized as follows. The mathematical model of a quadcopter UAV and problem description is presented in Section 2. In Section 3, the steps of constructing a nonlinear robust backstepping controller based on ESDO and stability analysis are derived. A numerical simulation conducted and compared with other existing methods is given in Section 4. Final statements of this study are given in Section 5 through some conclusions.

## 2. Mathematical Model and Problem Description

In this section, the mathematical model of a quadcopter UAV is constructed in the presence of external disturbances:

There are some assumptions are given as follows:

**Hypothesis** **1.** *The structure of a quadcopter UAV is considered as a rigid body*.

**Hypothesis** **2.** *The coefficients of four rotors and parameters of propellers are identical*.

**Figure 1:**
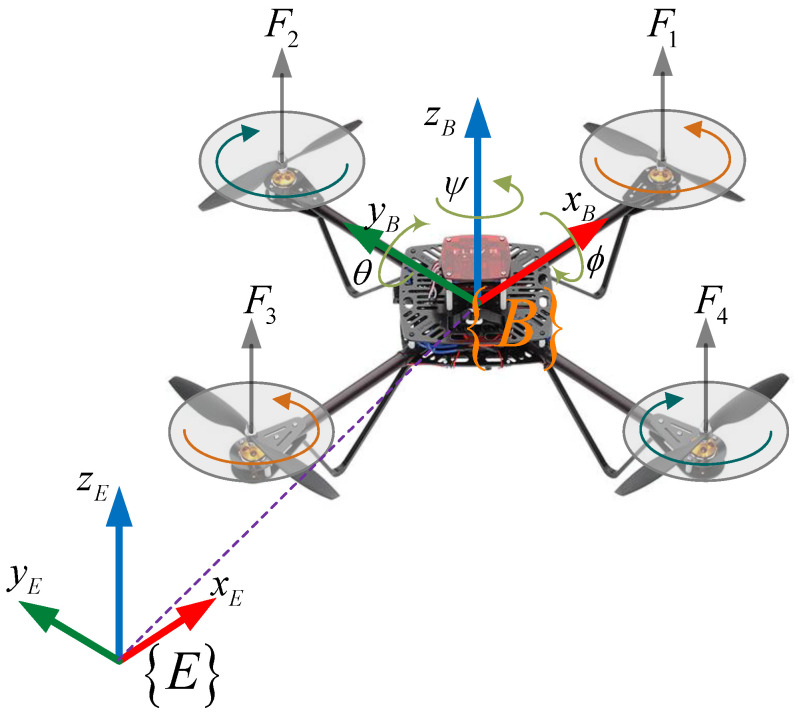
Quadcopter configuration.

The quadcopter UAV can be considered as a 6-DOFs rigid body, which is an idealized expression of invariable volume. Thus, the behavior of the quadcopter (i.e., rotational motion and translational motion) can be described through the movement of a particle positioned in the UAV center of mass. Obviously, a dynamic model of the vehicle entirely depends on aerodynamic force and torque generated by the speed of propellers mounted on four rotors. Thus, it is necessary to consider that the properties of four rotors and propellers are identical to facilitate the formulation of a mathematical model. Through two essential coordinate frames, i.e., Earth frame {E}, and body frame {B} (as shown in Figure 1), the dynamical model of a quadcopter UAV can be described from two subsystems of translational motion (Q) and rotational motion (Θ) by using the Newton–Euler formula as follows:

(1) $$\left\{ \begin{array}{l} \underset{Translation}{\left(Q\right)}\;\;:\;\;\left\{ \begin{array}{l} \dot{Q}=\upsilon \\ m\dot{\upsilon }=-G+T_{B}^{E}\left(\Theta \right)F_{aero}+F_{dis} \end{array}\right.\\ \;\;\underset{Rotation}{\left(\Theta \right)}\;\;\;\;:\;\;\left\{ \begin{array}{l} \dot{\Theta }=R_{B}^{E}\left(\Theta \right)\varpi \\ I\dot{\varpi }=-\left(\varpi \times I\varpi \right)-M_{gyros}+M_{aero}+M_{dis} \end{array}\right. \end{array}\right.$$

where $Q={\left[x,y,z\right]}^{T}\in {\mathbb{R}}^{3}$ is the absolute position of the quadrotor UAV with respect to the earth frame {E}; $\Theta ={\left[\phi ,\theta ,\psi \right]}^{T}\in {\mathbb{R}}^{3}$, ϕ,θ∈(−π/2,π/2), represent a vector of Euler angles (i.e., the roll, pitch, and yaw); $G={\left[0,0,mg\right]}^{T}\in {\mathbb{R}}^{3}$ with $m\in {\mathbb{R}}^{+}$ and $g=9.81\;m/s^{2}$ denote the total mass of the vehicle and gravity acceleration constant, respectively; $\varpi \in {\mathbb{R}}^{3}$ is angular velocity; $I=diag\left[I_{x},I_{y},I_{z}\right]\in {\mathbb{R}}^{3\times 3}$ denotes the matrix of the inertial moment along *x*, *y*, and *z* axes; $F_{aero}={\left[0,0,f_{z}\right]}^{T}\in {\mathbb{R}}^{3}$ and $M_{aero}={\left[\tau_{\phi },\tau_{\theta },\tau_{\psi }\right]}^{T}\in {\mathbb{R}}^{3}$ are the vectors of aerodynamic force and torque generated by multi-rotors and propellers of the vehicle, in which $f_{z}[N]$ is the thrust force; $\tau_{\phi },\tau_{\theta },\tau_{\psi }\left[N.m\right]$ represent the roll, pitch, and yaw torques, respectively. These values can be computed by:

(2) $$\left(\begin{array}{c} f_{z}\\ \tau_{\phi }\\ \tau_{\theta }\\ \tau_{\psi } \end{array}\right)=\left(\begin{array}{c} \sum_{i=1}^{4}F_{i}\\ l\left(F_{2}-F_{4}\right)\\ l\left(-F_{1}+F_{3}\right)\\ \sum_{i=1}^{4}{\left(-1\right)}^{i}M_{i} \end{array}\right)=\left(\begin{array}{c} b\left(\Omega_{1}^{2}+\Omega_{2}^{2}+\Omega_{3}^{2}+\Omega_{4}^{2}\right)\\ lb\left(\Omega_{2}^{2}-\Omega_{4}^{2}\right)\\ lb\left(-\Omega_{1}^{2}+\Omega_{3}^{2}\right)\\ d\left(-\Omega_{1}^{2}+\Omega_{2}^{2}-\Omega_{3}^{2}+\Omega_{4}^{2}\right) \end{array}\right)$$

where $F_{i}=b\;\Omega_{i}^{2}$, and $M_{i}=d\;\Omega_{i}^{2},\;\;i=1,2,\ldots ,4$ represent the aerodynamic force and torque produced by the rotor and propeller *i-*th; $b,d\in {\mathbb{R}}^{+}$ are thrust and drag coefficients, respectively; $\;\Omega_{i}$ is the angular velocity of motor *i-*th; *l* is the arm length of the vehicle frame.

In addition, $T_{B}^{E}\left(\Theta \right)$ and $R_{B}^{E}\left(\Theta \right)$ represent the translational and rotational transformation matrices from the body frame {*B*} to earth frame {*E*}, respectively. These values can be computed by

(3) $$T_{B}^{E}\left(\Theta \right)=\left(\begin{array}{ccc} C_{\theta }C_{\psi } & S_{\phi }S_{\theta }C_{\psi }-C_{\phi }S_{\psi } & C_{\phi }S_{\theta }C_{\psi }+S_{\phi }S_{\psi }\\ C_{\theta }S_{\psi } & S_{\phi }S_{\theta }S_{\psi }+C_{\phi }C_{\psi } & C_{\phi }S_{\theta }S_{\psi }-S_{\phi }C_{\psi }\\ -S_{\theta } & S_{\phi }C_{\theta } & C_{\phi }C_{\theta } \end{array}\right)$$

and

(4) $$R_{B}^{E}\left(\Theta \right)=\left(\begin{array}{ccc} 1 & S_{\phi }T_{\theta } & C_{\phi }T_{\theta }\\ 0 & C_{\phi } & -S_{\phi }\\ 0 & S_{\phi }/C_{\theta } & C_{\phi }/C_{\theta } \end{array}\right)$$

where $S_{\left(•\right)}$, $C_{\left(•\right)}$, and $T_{\left(•\right)}$ represent the notations of sin(•), cos(•), and tan(•), respectively.

It is proposed to consider that the variations in the three Euler angles are unprogressive maneuvers, meaning that the rotational behaviors of the roll, pitch, and yaw angles are surrounding the origin. Thus, $R_{B}^{E}\left(\Theta \right)$ is closely equivalent to the identity matrix, i.e., $R_{B}^{E}\left(\Theta \right)=I_{3\times 3}$; $M_{gyros}$ denotes the gyroscopic torque generated by the total residual angular velocity of four rotors, i.e., $\bar{\Omega }=\sum_{i=1}^{4}{\left(-1\right)}^{i+1}\Omega_{i},\;\;i=1,\ldots ,4$, effecting on a rigid body of the vehicle, its value is given by:

(5) $$M_{gyros}=\sum_{i=1}^{4}\left(J_{r}\left(\varpi \times {\hat{e}}_{3}\right){\left(-1\right)}^{i+1}\Omega_{i}\right)$$

where ${\hat{e}}_{3}={\left[0,0,1\right]}^{T}$ is a unit vector along the *z*-axis.

In addition, in order to design an efficient control law for a quadcopter UAV, it cannot ignore the undesirable influence of disturbances and/or uncertainties on the dynamical system. Hence, $F_{dis}={\left[\xi_{x},\xi_{y},\xi_{z}\right]}^{T}$ and ${Μ}_{dis}={\left[\xi_{\phi },\xi_{\theta },\xi_{\psi }\right]}^{T}$ are considered as the effects of perturbations/uncertainties on the translational and rotational subsystems, respectively.

From Equations (1)–(5), the mathematical model of a quadcopter UAV can be clearly described by:

(6) $$\left\{ \begin{array}{l} \underset{Translation}{\left(Q\right)}:\left(\begin{array}{c} \ddot{x}\\ \ddot{y}\\ \ddot{z} \end{array}\right)=\left(\begin{array}{c} \left[C_{\phi }S_{\theta }C_{\psi }+S_{\phi }S_{\psi }\right]f_{z}/m\\ \left[C_{\phi }S_{\theta }S_{\psi }-S_{\phi }C_{\psi }\right]f_{z}/m\\ -g+\left[C_{\phi }C_{\theta }\right]f_{z}/m \end{array}\right)+\left(\begin{array}{c} \xi_{x}\\ \xi_{y}\\ \xi_{z} \end{array}\right)\\ \;\;\underset{Rotation}{\left(\Theta \right)}\;\;:\left(\begin{array}{c} \ddot{\phi }\\ \ddot{\theta }\\ \ddot{\psi } \end{array}\right)=\left(\begin{array}{c} \dot{\theta }\dot{\psi }\left(I_{y}-I_{z}\right)/I_{x}-J_{r}\dot{\theta }\;\bar{\Omega }/I_{x}\\ \dot{\phi }\dot{\psi }\left(I_{z}-I_{x}\right)/I_{y}+J_{r}\dot{\phi }\;\bar{\Omega }/I_{y}\\ \dot{\phi }\dot{\theta }\left(I_{x}-I_{y}\right)/I_{z} \end{array}\right)+\left(\begin{array}{c} \tau_{\phi }/I_{x}\\ \tau_{\theta }/I_{y}\\ \tau_{\psi }/I_{z} \end{array}\right)+\left(\begin{array}{c} \xi_{\phi }\\ \xi_{\theta }\\ \xi_{\psi } \end{array}\right) \end{array}\right.$$

In order to construct a robust backstepping controller-based ESDO for improving the tracking performance of a quadcopter UAV, an equivalent MIMO control system under the effects of disturbances and/or uncertainties can be described by a state-space model as follows,

(7) $$\ddot{Z}(t)=g(Z,t)+ϒu(t)+\xi (t)$$

where $Z\in {\mathbb{R}}^{6}$, $u\in {\mathbb{R}}^{6}$, and $\xi \in {\mathbb{R}}^{6}$ represent the vectors of system states, control input, and lumped perturbations and/or uncertainties, respectively. Its detailed descriptions can be given as follows,

$$\begin{array}{l} Z={\left[z_{1},z_{2},z_{3},z_{4},z_{5},z_{6}\right]}^{T}={\left[x,y,z,\phi ,\theta ,\psi \right]}^{T}\\ \ddot{Z}={\left[{\ddot{z}}_{1},{\ddot{z}}_{2},{\ddot{z}}_{3},{\ddot{z}}_{4},{\ddot{z}}_{5},{\ddot{z}}_{6}\right]}^{T}={\left[\ddot{x},\ddot{y},\ddot{z},\ddot{\phi },\ddot{\theta },\ddot{\psi }\right]}^{T}\\ u={\left[u_{1},u_{2},u_{3},u_{4},u_{5},u_{6}\right]}^{T}={\left[u_{x},u_{y},u_{z},\tau_{\phi },\tau_{\theta },\tau_{\psi }\right]}^{T}\\ \xi ={\left[\xi_{1},\xi_{2},\xi_{3},\xi_{4},\xi_{5},\xi_{6}\right]}^{T}={\left[\xi_{x},\xi_{y},\xi_{z},\xi_{\phi },\xi_{\theta },\xi_{\psi }\right]}^{T} \end{array}$$

and the terms of $g(Z,t)\in {\mathbb{R}}^{6}$ and $ϒ\in {\mathbb{R}}^{6\times 6}$ are given as follows:

(8) $$g\left(Z,t\right)=\left[\begin{array}{c} 0\\ 0\\ 0\\ \dot{\theta }\dot{\psi }a_{1}-a_{2}\dot{\theta }\;\bar{\Omega }\\ \dot{\phi }\dot{\psi }a_{3}+a_{4}\dot{\phi }\;\bar{\Omega }\\ \;\;\;\;\dot{\phi }\dot{\theta }a_{5} \end{array}\right],\;\;with\;\;\left\{ \begin{array}{l} a_{1}=\left(I_{y}-I_{z}\right)/I_{x}\\ a_{2}=J_{r}/I_{x}\\ a_{3}=\left(I_{z}-I_{x}\right)/I_{y}\\ a_{4}=J_{r}/I_{y}\\ a_{5}=\left(I_{x}-I_{y}\right)/I_{z} \end{array}\right.$$

(9) $$ϒ=\left[\begin{array}{cc} I_{3\times 3} & O_{3\times 3}\\ O_{3\times 3} & diag\left(B\right) \end{array}\right],\;B={\left[b_{1},b_{2},b_{3}\right]}^{T},\;with\;\;\left\{ \begin{array}{l} b_{1}=1/I_{x}\\ b_{2}=1/I_{y}\\ b_{3}=1/I_{z} \end{array}\right.$$

The objective of controller design is to derive a robust backstepping control law guaranteeing a good convergence of the output state, Z, to the desired trajectory $Z^{d}\in {\mathbb{R}}^{6}$, $Z^{d}={\left[z_{1}^{d},z_{2}^{d},z_{3}^{d},z_{4}^{d},z_{5}^{d},z_{6}^{d}\right]}^{T}={\left[x_{d},y_{d},z_{d},\phi_{d},\theta_{d},\psi_{d}\right]}^{T}$ in the appearance of uncertain parameters and/or external perturbations. It can be seen that the quadcopter UAV is an underactuated mechanical system because the vehicle has four control inputs $\left(f_{z},\tau_{\phi },\tau_{\theta },\tau_{\psi }\right)$ but there are six degrees of freedom to be controlled (x,y,z,ϕ,θ,ψ). Hence, in order to design a robust controller for a full dynamic system, the attitude control for Euler angles (ϕ,θ,ψ), and position control for the three dimensions (x,y,z) are simultaneously constructed by using two separated subsystems (i.e., rotational and translational dynamics). The attitude subsystem comprising roll, pitch, and yaw rotation is controlled by three torques $\tau_{\phi },\tau_{\theta },\tau_{\psi }$ (i.e., $u_{4},u_{5},u_{6}$); the vertical position (*z*) is controlled by a control input $u_{z}$ obtained from Equations (6)–(9) as follows:

(10) $$u_{z}=-g+\left(\cos\phi \cos\theta \right)f_{z}/m$$

Therefore, the aerodynamic force $f_{z}$ can be derived from the controller $u_{z}$ as follows,

(11) $$f_{z}=\frac{m\left(u_{z}+g\right)}{\cos\phi \cos\theta },\;\;\;\;\;\forall \phi ,\theta \in \left(-\pi /2,\pi /2\right)$$

The remaining states of the control system (i.e., horizontal positions *x* and *y*), are indirectly controlled through desired attitude states $\left(\phi_{d},\theta_{d},\psi_{d}\right)$ and vertical control force $f_{z}$ achieved from Equations (6)–(9) as follows:

(12) $$\left\{ \begin{array}{l} u_{x}=\left(\cos\phi_{d}\sin\theta_{d}\cos\psi_{d}+\sin\phi_{d}\sin\psi_{d}\right)f_{z}/m\\ u_{y}=\left(\cos\phi_{d}\sin\theta_{d}\sin\psi_{d}-\sin\phi_{d}\cos\psi_{d}\right)f_{z}/m \end{array}\right.$$

where $u_{x},u_{y},u_{z}$ (i.e., $u_{1},u_{2},u_{3}$) are designed controllers for translational behaviors along *x*, *y*, and *z* axes, respectively; the heading angle, $\psi_{d}$, is known parameters in advance.

In addition, $\phi_{d},\;\theta_{d}\in \left(-\pi /2,\pi /2\right)$ are desired attitude angles generated from the position controller term $u_{x},u_{y}$ from Equation (12), $\left(\phi_{d},\;\theta_{d}\right)$ can be computed as follows:

(13) $$\left\{ \begin{array}{l} \phi_{d}=\sin^{-1}\left[m\left(u_{x}\sin\psi_{d}-u_{y}\cos\psi_{d}\right)/f_{z}\right]\\ \theta_{d}=\sin^{-1}\left[m\left(u_{x}\cos\psi_{d}+u_{y}\sin\psi_{d}\right)/\left(f_{z}\cos\phi_{d}\right)\right] \end{array}\right.$$

## 3. Robust Backstepping Control-Based ESDO

A robust backstepping controller based on an extended state/disturbance observer for a quadcopter UAV is presented in this section in the following steps:

*Step 1*: ESDO is designed by using the second-order system to observe the unmeasured system states consisting of the effectiveness of disturbances/uncertainties on the whole control system.

*Step 2:* A full robust backstepping controller is derived by an integration of the approximated values from the proposed ESDO to reimburse the negative effects of uncertainties and/or external disturbances on a quadcopter UAV. In addition, a stability analysis is also fulfilled to demonstrate a good convergence of the output states to desired trajectories. During this procedure, the precise knowledge of the upper bounds of perturbations is not required.

### 3.1. Extended State/Disturbance Observer (ESDO)

This subsection investigates a method to observe the unmeasured states of the vehicle system including the lumped uncertainties and/or external disturbances. To derive an ESDO, a MIMO nonlinear system is obtained from Equation (7) as follows,

(14) $$\left\{ \begin{array}{l} \left[\begin{array}{l} \ddot{Z}\\ \dot{\xi } \end{array}\right]=\left[\begin{array}{c} g(Z,t)\\ O \end{array}\right]+\left[\begin{array}{c} \left[ϒu(t)\right]\\ O \end{array}\right]+\left[\begin{array}{l} \xi (t)\\ O \end{array}\right]+\left[\begin{array}{l} O\\ \dot{\xi }(t) \end{array}\right]\\ y(t)=C_{1}z(t) \end{array}\right.$$

It can be rewritten as the shorter form:

(15) $$\left\{ \begin{array}{l} \dot{z}(t)=A_{1}z(t)+B_{1}g(Z,t)+B_{1}ϒu(t)+E_{1}\dot{\xi }(t)\\ y(t)=C_{1}z(t) \end{array}\right.$$

where $z(t)={\left[\dot{Z},\xi \right]}^{T}\in {\mathbb{R}}^{12}$ denotes a new vector of output state and disturbance included; $A_{1},C_{1}\in {\mathbb{R}}^{12\times 12}$, $A_{1}=\left[\begin{array}{cc} O_{6\times 6} & I_{6\times 6}\\ O_{6\times 6} & O_{6\times 6} \end{array}\right]$, $C_{1}=\left[\begin{array}{cc} I_{6\times 6} & O_{6\times 6}\\ O_{6\times 6} & I_{6\times 6} \end{array}\right]$; $B_{1},E_{1}\in {\mathbb{R}}^{12\times 6}$, $B_{1}={\left[I_{6\times 6},O_{6\times 6}\right]}^{T}$, $E_{1}={\left[O_{6\times 6},I_{6\times 6}\right]}^{T}$ are constant matrices, and $\dot{\xi }(t)$ is derivative of the general lumped disturbance vector; $I_{6\times 6}$ and $O_{6\times 6}$ represent the identity and null matrices, respectively.

The time-varying extended state/disturbance observer for a quadcopter UAV is proposed as follows:

(16) $$\left\{ \begin{array}{l} \dot{\hat{z}}(t)=A_{1}\hat{z}(t)+B_{1}g(Z,t)+B_{1}ϒu(t)+\Gamma_{1}\left[y(t)-\hat{y}(t)\right]\\ \hat{y}(t)=C_{1}\hat{z}(t) \end{array}\right.$$

where $\hat{z}(t)={\left[\hat{\dot{Z}},\hat{\xi }\right]}^{T}\in {\mathbb{R}}^{12}$ is an estimate state vector of z(t); $\Gamma_{1}\in {\mathbb{R}}^{12\times 12}$ is an observer gain matrix given by $\Gamma_{1}=\left[\begin{array}{cc} \alpha_{6\times 6} & O_{6\times 6}\\ \beta_{6\times 6} & O_{6\times 6} \end{array}\right]$, with $\left\{ \begin{array}{l} \alpha_{6\times 6}=diag\left[\alpha_{11},\alpha_{1i},\ldots ,\alpha_{16}\right]\\ \beta_{6\times 6}=diag\left[\beta_{11},\beta_{1i},\ldots ,\beta_{16}\right] \end{array}\right.$, $\alpha_{1i},\beta_{1i}\in {\mathbb{R}}^{+}$, i=1,…,6. Let $\tilde{\chi }(t)={\left[\tilde{\dot{Z}},\tilde{\xi }\right]}^{T}\in {\mathbb{R}}^{12}$ is an approximate error computed by $\tilde{\chi }(t)=z(t)-\hat{z}(t)$ as follows:

(17) $$\tilde{\chi }(t)=\left[\begin{array}{c} \tilde{\dot{Z}}(t)\\ \tilde{\xi }(t) \end{array}\right]=\left[\begin{array}{c} \dot{Z}(t)-\hat{\dot{Z}}(t)\\ \xi (t)-\hat{\xi }(t) \end{array}\right]$$

The performance of $\tilde{y}(t)=y(t)-\hat{y}(t)=C_{1}\left(z(t)-\hat{z}(t)\right)=C_{1}\tilde{\chi }(t)$ is bounded when the approximation error $\tilde{\chi }(t)$ is bounded.

The stability of the proposed ESDO can be achieved through the observer error model obtained by Equations (15) and (16) as follows:

(18) $$\begin{array}{c} \dot{\tilde{\chi }}(t)=\underset{\Phi_{1}\in {\mathbb{R}}^{12\times 12}}{\underset{︸}{\left(A_{1}-\Gamma_{1}C_{1}\right)}}\tilde{\chi }(t)+E_{1}\dot{\xi }(t)\\ \dot{\tilde{\chi }}(t)=\Phi_{1}\tilde{\chi }(t)+E_{1}\dot{\xi }(t) \end{array}$$

where $\Phi_{1}=A_{1}-\Gamma_{1}C_{1}\in {\mathbb{R}}^{12\times 12}$.

Obviously, it is always feasible to select constant values of $\alpha_{1i},\beta_{1i}\in {\mathbb{R}}^{+},\;i=1,\ldots ,6$ such that the matrix $\Phi_{1}$ meets the Hurwitz stability theorem meaning that the eigenvalues of matrix $\Phi_{1}$, (i.e., $\lambda =-\lambda_{j}<0$, j=1,2,…,12), are arbitrarily located in the left half side of the complex plane (LHP). Thus,

(19) $$\left|\lambda I_{12\times 12}-\Phi_{1}\right|=\prod_{j=1}^{12}\left(\lambda +\lambda_{j}\right)=0,\;\;\lambda_{j}>0$$

**Theorem** **1.** *Let assumes that there always exist positive constants of* $\delta ,\sigma ,\varphi \in {\mathbb{R}}^{+}$*in such a way that the following expression always satisfies:*

(20) $$\left\{ \begin{array}{l} \sup_{t\in \left[t_{0},\infty \right)}\left\|\dot{\xi }(t)\right\|\leq \delta \\ \left\|e^{\Phi_{1}t}\right\|\leq \sigma e^{-\varphi t} \end{array}\right.\;$$

*where*$\lambda_{\max}\left\{\Phi_{1}\right\}=-\varphi <0$*is a maximum eigenvalue of the matrix *$\Phi_{1}$*. The observation error,*$\tilde{\chi }(t)$*given by Equation (17), is strongly convergent to a small ball containing the origin zero if the presented ESDO given in Equation (16) is selected to observe the system states and lumped perturbations of a quadcopter UAV.*

**Proof** **of** **Theorem** **1.** The solution, $\tilde{\chi }(t)$, of the differential Equation (18) can be easily achieved by [39] as follows:

(21) $$\tilde{\chi }(t)=e^{\Phi_{1}\left(t-t_{0}\right)}\tilde{\chi }(t_{0})+\int_{t_{0}}^{t}e^{\Phi_{1}\left(t-\tau \right)}E_{1}\dot{\xi }(\tau )d\tau$$

The inequality, $\left\|e^{\Phi_{1}\left(t-\tau \right)}E_{1}\dot{\xi }(\tau )\right\|\leq \left\|e^{\Phi_{1}\left(t-\tau \right)}\right\|\left\|E_{1}\right\|\left\|\dot{\xi }(\tau )\right\|\leq \sigma \delta \left\|E_{1}\right\|e^{-\varphi \left(t-\tau \right)}$, is always satisfied with the assumption given in Equation (20). In addition, the term of $\sigma \delta \left\|E_{1}\right\|e^{-\varphi \left(t-\tau \right)}$ is a positive scalar: thus, the bound of approximate error, $\tilde{\chi }(t)$, can be obtained as follows:

(22) $$\begin{array}{l} \left\|\tilde{\chi }(t)\right\|\leq \left\|e^{\Phi_{1}\left(t-t_{0}\right)}\right\|\left\|\tilde{\chi }(t_{0})\right\|+\left\|\int_{t_{0}}^{t}e^{\Phi_{1}\left(t-\tau \right)}E_{1}\dot{\xi }(\tau )d\tau \right\|\\ \;\;\;\;\;\;\;\;\;\;\;\;\;\;\leq \sigma e^{-\varphi \left(t-t_{0}\right)}\left\|\tilde{\chi }(t_{0})\right\|+\int_{t_{0}}^{t}\left\|e^{\Phi_{1}\left(t-\tau \right)}E_{1}\dot{\xi }(\tau )\right\|d\tau \\ \;\;\;\;\;\;\;\;\;\;\;\;\;\;\leq \sigma e^{-\varphi \left(t-t_{0}\right)}\left\|\tilde{\chi }(t_{0})\right\|+\sigma \delta \left\|E_{1}\right\|\int_{t_{0}}^{t}e^{-\varphi \left(t-\tau \right)}d\tau \\ \;\;\;\;\;\;\;\;\;\;\;\;\;\;\leq \sigma e^{-\varphi \left(t-t_{0}\right)}\left\|\tilde{\chi }(t_{0})\right\|+\sigma \delta \left\|E_{1}\right\|\frac{e^{-\varphi t}}{\varphi }\int_{t_{0}}^{t}e^{\varphi \tau }d\left(\varphi \tau \right)\\ \;\;\;\;\;\;\;\;\;\;\;\;\;\;\leq \sigma e^{-\varphi \left(t-t_{0}\right)}\left\|\tilde{\chi }(t_{0})\right\|+\sigma \delta \frac{\left\|E_{1}\right\|}{\varphi }\left(1-e^{-\varphi \left(t-t_{0}\right)}\right) \end{array}$$

Due to $e^{-\varphi \left(t-t_{0}\right)}\to 0,\;\;\;t_{0}\;\leq \forall t\to \infty$, thus $\tilde{\chi }(t)$ is bounded by:

(23) $$\left\|\tilde{\chi }(t)\right\|\leq \frac{\delta \sigma \left\|E_{1}\right\|}{\varphi }=\mu ,\;\;\;\mu >0$$

The proof of Theorem 1 is completed. □

**Remark** **1.** *From the result in Equation (23), we can conclude that the observation error,* $\left\|\tilde{\chi }(t)\right\|$ *, is ultimately bounded by a constant* μ *, if the observation gains* $\alpha_{1i},\beta_{1i}\in {\mathbb{R}}^{+},i=1,\ldots ,6$ *are properly chosen such that matrix* $\Phi_{1}$ *satisfies the Hurwitz criteria. Therefore,* $\left\|\tilde{\dot{Z}}(t)\right\|$ *and* $\left\|\tilde{\xi }(t)\right\|$ *are also convergent to a small region containing the origin zero, i.e.,*

(24) $$\left\|\tilde{\dot{Z}}(t)\right\|\leq \mu ,\;\;\left\|\tilde{\xi }(t)\right\|\leq \mu$$

### 3.2. Robust Backstepping Controller Design

**Figure 2:**
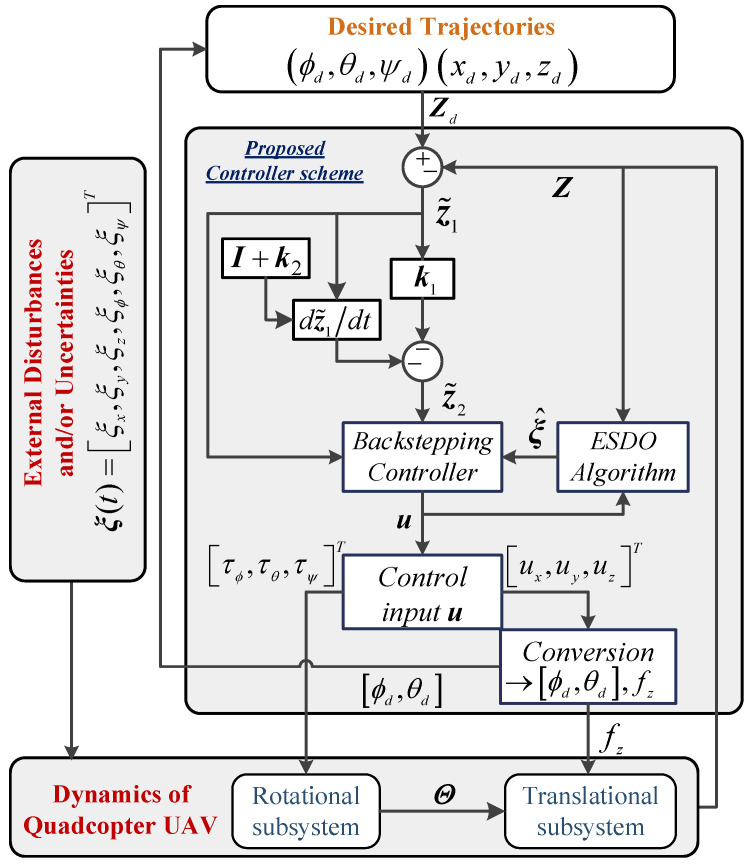
General scheme of UAV’s control system.

In this subsection, a robust backstepping controller is constructed by a combination of the approximated value from the proposed ESDO above and backstepping control technique to reimburse the negative effects of uncertainties and/or external disturbances on a quadcopter UAV. The presented controller guarantees the convergence of the output state, $Z={\left[x,y,z,\phi ,\theta ,\psi \right]}^{T}$, to the desired trajectory $Z^{d}={\left[x_{d},y_{d},z_{d},\phi_{d},\theta_{d},\psi_{d}\right]}^{T}$. A general scheme of the proposed controller for a quadcopter UAV is shown in Figure 2. Let ${\tilde{z}}_{1}={\left[{\tilde{z}}_{11},{\tilde{z}}_{1i},\ldots ,{\tilde{z}}_{16}\right]}^{T}\in {\mathbb{R}}^{6},\;i=1,2,\ldots ,6$ defines a vector of real tracking error

(25) $$\begin{array}{l} {\tilde{z}}_{1}=Z^{d}-Z\\ \;\;\;\;={\left[x_{d}-x,y_{d}-y,z_{d}-z,\phi_{d}-\phi ,\theta_{d}-\theta ,\psi_{d}-\psi \right]}^{T} \end{array}$$

In order to derive a backstepping controller, an auxiliary desired state, $Z_{aux}\in {\mathbb{R}}^{6}$, used for a virtual control system, is presented as follows:

(26) $$Z_{aux}=Z^{d}+k_{1}\int_{0}^{t}{\tilde{z}}_{1}dt+k_{2}{\tilde{z}}_{1}$$

where $k_{1}=diag(k_{11},k_{1i},\ldots ,k_{16}),\;\;k_{2}=diag(k_{21},k_{2i},\ldots ,k_{26})$, $k_{1i},k_{2i}\in {\mathbb{R}}^{+},i=1,\ldots ,6$ are diagonal matrices of controller gains.

Time derivative the auxiliary state is computed from Equation (26) as follows:

(27) $${\dot{Z}}_{aux}={\dot{Z}}^{d}+k_{1}{\tilde{z}}_{1}+k_{2}{\dot{\tilde{z}}}_{1}$$

Let ${\tilde{z}}_{2}={\left[{\tilde{z}}_{21},{\tilde{z}}_{2i},\ldots ,{\tilde{z}}_{26}\right]}^{T}\in {\mathbb{R}}^{6}$ denote a vector of virtual tracking error computed by the following equation

(28) $$\begin{array}{l} {\tilde{z}}_{2}=\dot{Z}-{\dot{Z}}_{aux}\\ \;\;\;\;=\dot{Z}-{\dot{Z}}^{d}-k_{1}{\tilde{z}}_{1}-k_{2}{\dot{\tilde{z}}}_{1} \end{array}$$

From Equations (25) and (28), it can be seen that

(29) $$\begin{array}{l} {\dot{\tilde{z}}}_{1}={\dot{Z}}^{d}-\dot{Z}\\ \;\;\;\;=-{\tilde{z}}_{2}-k_{1}{\tilde{z}}_{1}-k_{2}{\dot{\tilde{z}}}_{1}\\ \;\;\;\;=\underset{\Psi \in {\mathbb{R}}^{6\times 6}}{\underset{︸}{{\left(I_{6\times 6}+k_{2}\right)}^{-1}}}\left(-{\tilde{z}}_{2}-k_{1}{\tilde{z}}_{1}\right)\\ \;\;\;\;=-\Psi \left({\tilde{z}}_{2}+k_{1}{\tilde{z}}_{1}\right) \end{array}$$

where $\Psi \in {\mathbb{R}}^{6\times 6}$ is positive diagonal matrix given by,

(30) $$\begin{array}{l} \Psi ={\left(I_{6\times 6}+k_{2}\right)}^{-1}\\ \;\;\;\;=diag\left(\frac{1}{1+k_{21}},\frac{1}{1+k_{2i}},\ldots ,\frac{1}{1+k_{26}}\right),\;\;\;i=1,2,\ldots ,6 \end{array}$$

Substituting Equations (28) and (29), the virtual tracking error ${\tilde{z}}_{2}$ is computed by:

(31) $$\begin{array}{l} {\tilde{z}}_{2}=\dot{Z}-{\dot{Z}}^{d}-k_{1}{\tilde{z}}_{1}+k_{2}\Psi \left({\tilde{z}}_{2}+k_{1}{\tilde{z}}_{1}\right)\\ \;\;\;\;=\underset{P\in {\mathbb{R}}^{6\times 6}}{\underset{︸}{{\left(I_{6\times 6}-k_{2}\Psi \right)}^{-1}}}\left(\dot{Z}-{\dot{Z}}^{d}\right)-k_{1}{\tilde{z}}_{1}\\ \;\;\;\;=P\left(\dot{Z}-{\dot{Z}}^{d}\right)-k_{1}{\tilde{z}}_{1} \end{array}$$

where $P\in {\mathbb{R}}^{6\times 6}$ is positive diagonal matrix given by:

(32) $$\begin{array}{l} P={\left(I_{6\times 6}-k_{2}\Psi \right)}^{-1}\\ \;\;\;\;=diag\left(1+k_{21},1+k_{2i},\ldots ,1+k_{26}\right),\;\;\;\;i=1,2,\ldots ,6 \end{array}$$

The time derivative of ${\tilde{z}}_{2}$ is obtained from Equations (7) and (31) as follows,

(33) $$\begin{array}{l} {\dot{\tilde{z}}}_{2}=P\left(\ddot{Z}-{\ddot{Z}}^{d}\right)-k_{1}{\dot{\tilde{z}}}_{1}\\ \;\;\;\;=P\left[g(Z,t)+ϒu(t)+\xi (t)-{\ddot{Z}}^{d}\right]-k_{1}{\dot{\tilde{z}}}_{1} \end{array}$$

In order to stabilize the attitude control and also guarantee a good trajectory tracking of position control for a quadcopter UAV, the proposed robust backstepping controller-based ESDO, u(t), can be designed as the following equation,

(34) $$u={ϒ}^{-1}\left({\ddot{Z}}^{d}-g(Z,t)+\left(I_{6\times 6}-k_{2}\Psi \right)k_{1}{\dot{\tilde{z}}}_{1}-\hat{\xi }(t)+\left(I_{6\times 6}-k_{2}\Psi \right)\Psi^{T}{\tilde{z}}_{1}-k_{3}{\tilde{z}}_{2}\right)$$

where $k_{3}=diag\left(k_{31},k_{3i},\ldots ,k_{36}\right)\in {\mathbb{R}}^{6\times 6},\;\;k_{3i}>0,\;\;i=1,2,\ldots ,6$.

The stability analysis of the control system by using the controller u(t) is presented in the next section.

### 3.3. Stability Analysis of the Proposed Robust Backstepping Controller

The stability analysis of the control system is presented in this subsection as follows,

**Lemma** **1** ([39])**.** *Let* h(t),W(t):[0,∞)↦ℝ*. Then*

(35) $$\dot{W}(t)\leq -\varepsilon W(t)+h(t),\;\;\;\;\forall t\geq t_{0}\geq 0$$

*implies that*

(36) $$W(t)\leq e^{-\varepsilon \left(t-t_{0}\right)}W\left(t_{0}\right)+\int_{t_{0}}^{t}e^{-\varepsilon \left(t-\tau \right)}h\left(\tau \right)d\tau ,\;\;\;\;\forall t\geq t_{0}\geq 0$$

*for any finite constant* ε.

**Proof** **of** **Lemma** **1.** Let $\vartheta (t)≜\dot{W}(t)+\varepsilon W(t)-h(t)$, $\;\forall t\geq t_{0}\geq 0$ defines a new auxiliary variable. From the expression (35) can be derived ϑ(t)≤0, and $\dot{W}(t)$ is also obtained by,

(37) $$\dot{W}(t)=-\varepsilon W(t)+h(t)+\vartheta (t)$$

The solution, W(t), of differential Equation (37) can be achieved by [39] as follows

(38) $$W(t)\;=\;e^{-\varepsilon \left(t-t_{0}\right)}W\left(t_{0}\right)\;+\;\int_{t_{0}}^{t}e^{-\varepsilon \left(t-\tau \right)}h\left(\tau \right)d\tau \;+\;\int_{t_{0}}^{t}e^{-\varepsilon \left(t-\tau \right)}\vartheta \left(\tau \right)d\tau$$

$$\overset{\vartheta \left(t\right)\leq 0,\;\;\;\forall t\geq t_{0}\geq 0}{\to }W(t)\leq e^{-\varepsilon \left(t-t_{0}\right)}W\left(t_{0}\right)+\int_{t_{0}}^{t}e^{-\varepsilon \left(t-\tau \right)}h\left(\tau \right)d\tau$$

The proof of Lemma 1 is completed. □

**Theorem** **2.** *Consider the disturbed dynamic system (7). The proposed controller presented in Equation (34) can guarantee the ultimate convergence of the system output states of a quadcopter UAV to desired trajectories, i.e., the trajectory tracking errors* ${\tilde{z}}_{1},{\tilde{z}}_{2}$*are bounded by a small region neighborhood of zero.*

**Proof** **of** **Theorem** **2.** A Lyapunov function candidate for the trajectory tracking error is chosen by,

(39) $$\begin{array}{l} V\left({\tilde{z}}_{1},{\tilde{z}}_{2}\right)=\frac{1}{2}{\tilde{z}}_{1}^{T}{\tilde{z}}_{1}+\frac{1}{2}{\tilde{z}}_{2}^{T}{\tilde{z}}_{2}\\ \;\;\;\;\;\;\;\;\;\;\;\;\;\;\;\;\;\;\;\;=\frac{1}{2}\sum_{i=1}^{6}\left({\tilde{z}}_{1i}^{2}+{\tilde{z}}_{2i}^{2}\right) \end{array}$$

It is easy to achieve the first-time derivative of the function $V\left({\tilde{z}}_{1},{\tilde{z}}_{2}\right)$ presented in Equation (39) from Equations (29) and (33) as follows,

(40) $$\begin{array}{l} \dot{V}\left({\tilde{z}}_{1},{\tilde{z}}_{2}\right)={\tilde{z}}_{1}^{T}{\dot{\tilde{z}}}_{1}+{\tilde{z}}_{2}^{T}{\dot{\tilde{z}}}_{2}\\ \;\;\;\;\;\;\;\;\;\;\;\;\;\;\;\;\;=-{\tilde{z}}_{1}^{T}\Psi \left({\tilde{z}}_{2}+k_{1}{\tilde{z}}_{1}\right)+{\tilde{z}}_{2}^{T}\left(P\left[g(Z,t)+ϒu(t)+\xi (t)-{\ddot{Z}}^{d}\right]-k_{1}{\dot{\tilde{z}}}_{1}\right) \end{array}$$

Substituting the proposed controller, u(t), given in Equation (34), and matrix ***P*** in Equation (32) into Equation (40) and doing some mathematical conversion steps, the value of $\dot{V}\left({\tilde{z}}_{1},{\tilde{z}}_{2}\right)$ can be obtained as follows,

(41) $$\begin{array}{l} \dot{V}\left({\tilde{z}}_{1},{\tilde{z}}_{2}\right)=-{\tilde{z}}_{1}^{T}\Psi \left({\tilde{z}}_{2}+k_{1}{\tilde{z}}_{1}\right)+{\tilde{z}}_{2}^{T}\left(\Psi^{T}{\tilde{z}}_{1}-Pk_{3}{\tilde{z}}_{2}+P\left(\xi (t)-\hat{\xi }(t)\right)\right)\\ \;\;\;\;\;\;\;\;\;\;\;\;\;\;\;\;\;=-{\tilde{z}}_{1}^{T}\Psi {\tilde{z}}_{2}-{\tilde{z}}_{1}^{T}\underset{M\in {\mathbb{R}}^{6\times 6}}{\underset{︸}{\left(\;\Psi k_{1}\right)}}{\tilde{z}}_{1}+{\tilde{z}}_{2}^{T}\Psi^{T}{\tilde{z}}_{1}-{\tilde{z}}_{2}^{T}\underset{N\in {\mathbb{R}}^{6\times 6}}{\underset{︸}{\left(\;Pk_{3}\right)}}{\tilde{z}}_{2}+{\tilde{z}}_{2}^{T}P\tilde{\xi }\\ \;\;\;\;\;\;\;\;\;\;\;\;\;\;\;\;\;=-{\tilde{z}}_{1}^{T}M{\tilde{z}}_{1}-{\tilde{z}}_{2}^{T}N{\tilde{z}}_{2}+{\tilde{z}}_{2}^{T}P\tilde{\xi } \end{array}$$

where $M,N\in {\mathbb{R}}^{6\times 6}$ are positive diagonal constant matrices, its values can be computed from Equations (30) and (32) as follows,

(42) $$\begin{array}{l} M=\Psi k_{1}\\ \;\;\;\;\;=diag\left(\frac{k_{11}}{1+k_{21}},\frac{k_{1i}}{1+k_{2i}},\ldots ,\frac{k_{16}}{1+k_{26}}\right),\;\;\;i=1,2,\ldots ,6 \end{array}$$

(43) $$\begin{array}{l} N=Pk_{3}={\left(I_{6\times 6}-k_{2}\Psi \right)}^{-1}k_{3}\\ \;\;\;\;=diag\left(k_{31}\left(1+k_{21}\right),k_{3i}\left(1+k_{2i}\right),\ldots ,k_{36}\left(1+k_{26}\right)\right),\;\;i=1,2,\ldots ,6 \end{array}$$

In addition, the values of ${\tilde{z}}_{1}^{T}M{\tilde{z}}_{1}$, ${\tilde{z}}_{2}^{T}N{\tilde{z}}_{2}$, and ${\tilde{z}}_{2}^{T}P\tilde{\xi }$ from Equation (41) can be computed from Equations (32), (42), and (43) as follows,

(44) $${\tilde{z}}_{1}^{T}M{\tilde{z}}_{1}=\sum_{i=1}^{6}\frac{k_{1i}}{1+k_{2i}}{\tilde{z}}_{1i}^{2}$$

(45) $${\tilde{z}}_{2}^{T}N{\tilde{z}}_{2}=\sum_{i=1}^{6}k_{3i}\left(1+k_{2i}\right){\tilde{z}}_{2i}^{2}$$

(46) $${\tilde{z}}_{2}^{T}P\tilde{\xi }=\sum_{i=1}^{6}\left(1+k_{2i}\right){\tilde{z}}_{2i}{\tilde{\xi }}_{i}$$

Using Young’s inequality for $\left|{\tilde{z}}_{2i}\right|$ and $\left|{\tilde{\xi }}_{i}\right|$, the following expression is always satisfied,

(47) $${\tilde{z}}_{2i}{\tilde{\xi }}_{i}\leq \left|{\tilde{z}}_{2i}\right|\left|{\tilde{\xi }}_{i}\right|\leq \frac{1}{2}{\tilde{z}}_{2i}^{2}+\frac{1}{2}{\tilde{\xi }}_{i}^{2}$$

From Equations (44)–(47), the derivative function $\dot{V}\left({\tilde{z}}_{1},{\tilde{z}}_{2}\right)$ given in Equation (41) can be obtained as follows,

(48) $$\begin{array}{l} \dot{V}\left({\tilde{z}}_{1},{\tilde{z}}_{2}\right)=-{\tilde{z}}_{1}^{T}M{\tilde{z}}_{1}-{\tilde{z}}_{2}^{T}N{\tilde{z}}_{2}+{\tilde{z}}_{2}^{T}P\tilde{\xi }\\ \;\;\;\;\;\;\;\;\;\;\;\;\;\;\;\;\leq -\sum_{i=1}^{6}\left(\frac{k_{1i}}{1+k_{2i}}{\tilde{z}}_{1i}^{2}\right)-\sum_{i=1}^{6}\left(k_{3i}\left(1+k_{2i}\right){\tilde{z}}_{2i}^{2}\right)+\frac{1}{2}\sum_{i=1}^{6}\left[\left(1+k_{2i}\right)\left({\tilde{z}}_{2i}^{2}+{\tilde{\xi }}_{i}^{2}\right)\right]\\ \;\;\;\;\;\;\;\;\;\;\;\;\;\;\;\;\leq -\sum_{i=1}^{6}\left(\frac{k_{1i}}{1+k_{2i}}{\tilde{z}}_{1i}^{2}\right)-\sum_{i=1}^{6}\left[k_{3i}\left(1+k_{2i}\right){\tilde{z}}_{2i}^{2}\right]+\frac{1}{2}\sum_{i=1}^{6}\left[\left(1+k_{2i}\right){\tilde{z}}_{2i}^{2}\right]\;+\frac{1}{2}\sum_{i=1}^{6}\left[\left(1+k_{2i}\right){\tilde{\xi }}_{i}^{2}\right]\\ \;\;\;\;\;\;\;\;\;\;\;\;\;\;\;\;\leq -\sum_{i=1}^{6}\left(\frac{k_{1i}}{1+k_{2i}}{\tilde{z}}_{1i}^{2}\right)-\sum_{i=1}^{6}\left[\left(k_{3i}-\frac{1}{2}\right)\left(1+k_{2i}\right){\tilde{z}}_{2i}^{2}\right]+\frac{1}{2}\sum_{i=1}^{6}\left[\left(1+k_{2i}\right){\tilde{\xi }}_{i}^{2}\right] \end{array}$$

Let $\rho ,\eta \in {\mathbb{R}}^{+}$ be positive constants, which always satisfy the following conditions,

(49) $$\left\{ \begin{array}{l} \rho =\min\left\{\frac{k_{1i}}{1+k_{2i}},\;\left(k_{3i}-\frac{1}{2}\right)\left(1+k_{2i}\right)\right\}\\ \eta =\max\left\{1+k_{2i}\right\} \end{array}\right.,\;\;\;i=1,2,\ldots ,6$$

Hence, from Equations (24), (39) and (47)–(49), the below inequality always satisfies,

(50) $$\begin{array}{l} \dot{V}\leq -\rho \sum_{i=1}^{6}{\tilde{z}}_{1i}^{2}-\rho \sum_{i=1}^{6}{\tilde{z}}_{2i}^{2}+\frac{1}{2}\eta \sum_{i=1}^{6}{\tilde{\xi }}_{i}^{2}\\ \;\;\;\leq -\rho \sum_{i=1}^{6}\left({\tilde{z}}_{1i}^{2}+{\tilde{z}}_{2i}^{2}\right)+\frac{1}{2}\eta \sum_{i=1}^{6}{\tilde{\xi }}_{i}^{2}\\ \;\;\;\leq -2\rho V+\frac{1}{2}\eta {\left\|\tilde{\xi }\right\|}^{2}\\ \;\;\;\leq -2\rho V+\frac{1}{2}\eta \mu^{2} \end{array}$$

According to Lemma 1, the solution V(t) for inequality (50) can be obtained as follows,

(51) $$\begin{array}{l} V(t)\leq e^{-2\rho \left(t-t_{0}\right)}V\left(t_{0}\right)+\frac{1}{2}\eta \mu^{2}\int_{t_{0}}^{t}e^{-2\rho \left(t-\tau \right)}d\tau \\ \;\;\;\;\;\;\;=e^{-2\rho \left(t-t_{0}\right)}V\left(t_{0}\right)+\frac{1}{2}\eta \mu^{2}e^{-2\rho t}\int_{t_{0}}^{t}e^{2\rho \tau }d\tau \\ \;\;\;\;\;\;\;=e^{-2\rho \left(t-t_{0}\right)}V\left(t_{0}\right)+\frac{\eta \mu^{2}e^{-2\rho t}}{4\rho }\left(e^{2\rho t}-e^{2\rho t_{0}}\right)\\ \;\;\;\;\;\;\;=e^{-2\rho \left(t-t_{0}\right)}V\left(t_{0}\right)+\frac{\eta \mu^{2}}{4\rho }\left(1-e^{-2\rho \left(t-t_{0}\right)}\right) \end{array}$$

From Equation (51), it is obvious, when t→+∞, the function V(t) is constantly convergent by

(52) $$V\left(\infty \right)\leq \frac{\eta \mu^{2}}{4\rho }$$

Theorem 2 is completely proven. □

**Remark** **2.** *From the result in Equation (52), we can conclude that the function *$V\left({\tilde{z}}_{1},{\tilde{z}}_{2}\right)$*is bounded when* t→+∞*, and its value converges to *$\eta \mu^{2}/\left(4\rho \right)$*. It implies that the trajectory tracking errors *${\tilde{z}}_{1},{\tilde{z}}_{2}$*are convergent to a small region containing the origin zero.*

## 4. Simulation Results and Discussions

In this Section, a numerical simulation is carried out to verify the effectiveness of the proposed Robust Backstepping Control based on Extended State/Disturbance Observer for a quadcopter UAV through a scenario of trajectory tracking test, where different types of external perturbations and parametric uncertainties are considered. A comparative flight simulation between the proposed controller and other existent algorithms such as Sliding Mode Control [26] and Active Disturbance Rejection Control (ADRC) [38] is conducted on the same vehicle model with an identical working environment to emphasize the superior performance of the proposed method. The physical system parameters of a quadcopter UAV and the controller coefficients to perform a numerical simulation are provided in Table 1 and Table 2, respectively, in which the controller parameters given in Table 2 are selected to ensure the rapid response of the UAV system and minor trajectory tracking error.

A scenario of numerical simulation can be described as follows: the quadcopter UAV starts to takeoff from an initial state $Z_{0}={\left[x_{0},y_{0},z_{0},\phi_{0},\theta_{0},\psi_{0}\right]}^{T}={\left[8,0,0,0,0,0.1\right]}^{T}$. A control mission of the vehicle is to drive the UAV’s position closely tracking a typical desired trajectory given by Equation (53). During the working process, the flight of the quadcopter is influenced by randomly lumped perturbations.

(53) $$\left\{ \begin{array}{l} x_{d}=5+5\cos t/\left(1+\sin^{2}t\right)\\ y_{d}=5\sin t\cos t/\left(1+\sin^{2}t\right)\\ z_{d}=2 \end{array}\right.$$

**Figure 3:**
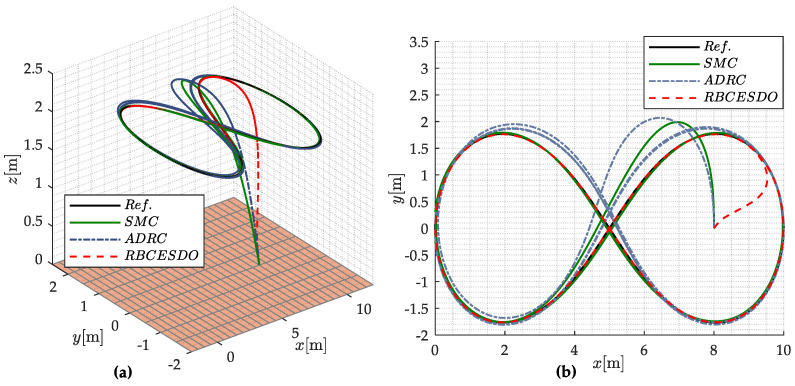
Performance comparison of SMC, ADRC, and RBCESDO in both 3D (**a**) and 2D (**b**) trajectory tracking tests on a quadcopter UAV.

**Figure 4:**
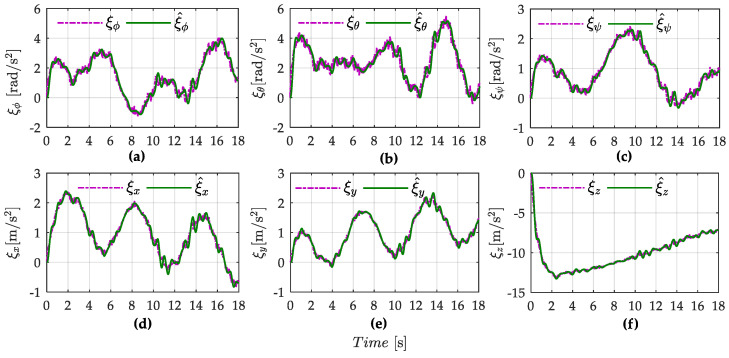
Disturbance estimation in attitude dynamic (**a**–**c**) and position dynamic (**d**–**f**) of the proposed ESDO.

**Figure 5:**
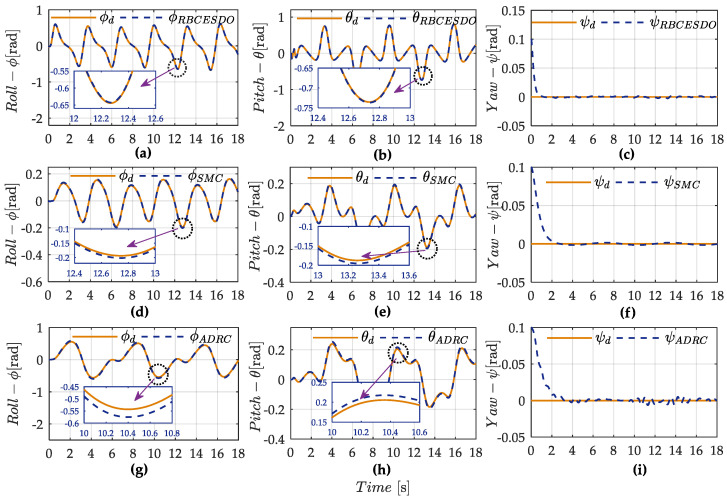
Comparison of attitude performance between the proposed RBCESDO (**a**–**c**), SMC (**d**–**f**), and ADRC (**g**–**i**).

**Figure 6:**
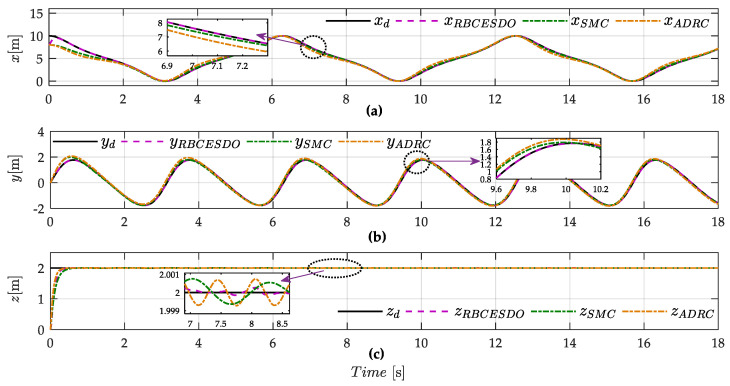
Comparison of position performance between the proposed RBCESDO, SMC, and ADRC: horizontal position *x* (**a**), *y* (**b**), and vertical position *z* (**c**).

From the simulation result shown in Figure 3a,b, the tracking performance of the proposed RBCESDO is significantly better than that of the Sliding Mode Control [26] and Active Disturbance Rejection Control [38] in both two-dimensional (2D) and three-dimensional (3D) trajectories in general. According to Figure 3a, the RBCESDO algorithm exhibits a rapid altitude achievement (*z* = 2 m). In addition, the horizontal position tracking (*x* and *y*) also responds faster and more accurately than the other considered methods. As a result, the vehicle position rapidly tracks the desired trajectory at *x* = 9 m, *y* = 1.5 m, while the corresponding position is slowly convergent by using SMC and ADRC because of the powerfully approximate capacity of the proposed extended state/disturbance observer. Following the result shown in Figure 4, the undesirable lumped perturbations’ influence on the attitude dynamic $\left(\xi_{\phi },\;\xi_{\theta },\;\xi_{\psi }\right)$ and on the position dynamic $\left(\xi_{x},\;\xi_{y},\;\xi_{z}\right)$ are closely estimated by $\left({\hat{\xi }}_{\phi },\;{\hat{\xi }}_{\theta },\;{\hat{\xi }}_{\psi }\right)$ and $\left({\hat{\xi }}_{x},\;{\hat{\xi }}_{y},\;{\hat{\xi }}_{z}\right)$, respectively. These approximated values are integrated with the proposed backstepping controller given in Equation (34) to compensate for the effects of disturbances/uncertainties. Thus, the performance of attitude states (roll, pitch, and yaw angle) and position states (*x, y,* and *z*) of the proposed RBCESDO is more precisely convergent to the reference trajectories compared with other methods as shown in Figure 5a–c and Figure 6.

**Figure 7:**
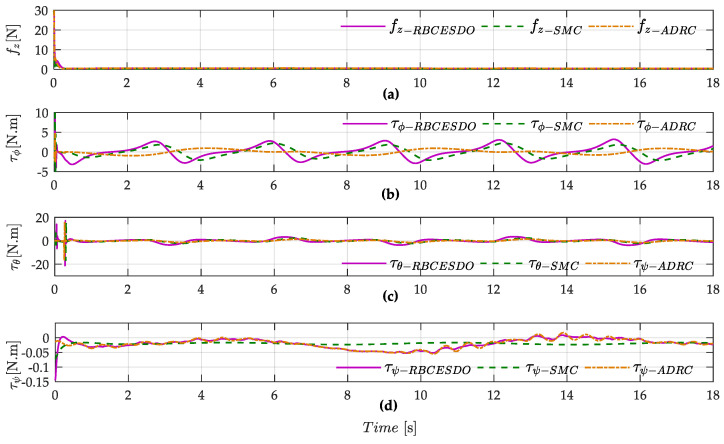
Comparison of the controller performance between RBCESDO, SMC, and ADRC: vertical force (**a**), roll torque (**b**), pitch torque (**c**) and yaw torque (**d**).

On the other hand, a general advantage of all three comparative methods (i.e., SMC, ADRC, and RBCESDO) is to eliminate the chattering effect of the controller, as shown in Figure 7. However, to remove the chattering phenomenon in the SMC technique, the high gain parameters of the switching law must be avoided. Thus, the SMC controller cannot be strictly guaranteed in both a tracking precision of the quadcopter UAV to a reference path and a strong anti-chattering effect. In this simulation, the moderate switching controller gains of the SMC method are selected instead of the large ones to ensure the anti-chattering capacity, resulting in the larger tracking errors of roll, pitch, and yaw angles, and larger error of positions are generated in comparison with the proposed RBCESDO method (as shown in Figure 5d–f for attitude control and Figure 6 for position control), while these fluctuation errors are more amplified with ADRC control algorithm as exhibited in Figure 5g–i and Figure 6.

**Figure 8:**
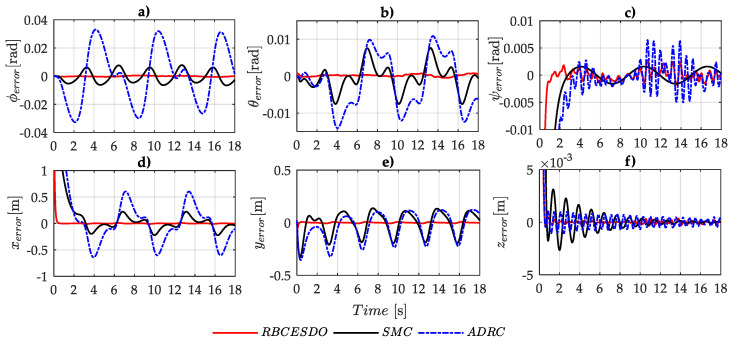
Comparison of attitude tracking error (**a**–**c**) and position tracking error (**d**–**f**) between the proposed RBCESDO, SMC, and ADRC.

**Figure 9:**
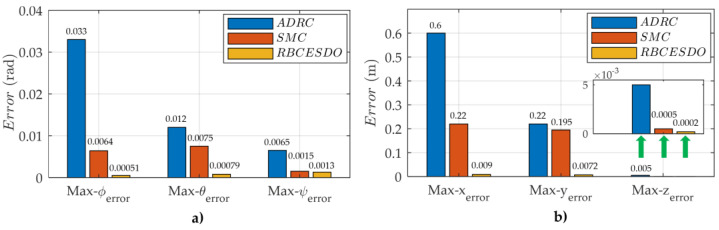
Comparison of maximum attitude tracking error (**a**) and maximum position tracking error (**b**) of the proposed RBCESDO, SMC, and ADRC.

To emphasize a significantly superior performance of the proposed controller compared to the other algorithms, a convergence to zero of the attitude tracking error $\left(\phi_{error},\theta_{error},\psi_{error}\right)$ and position tracking error $\left(x_{error},y_{error},z_{error}\right)$ of the three considered methods (RBCESDO, SMC, and ADRC) is also portrayed in Figure 8 and Figure 9, which exhibit that the proposed controller can obtain the best performance with the smallest tracking error and fastest response. More specifically, the maximum of the attitude tracking error of the RBCESDO reaches about $\mathrm{Max}\{\phi_{error}\}\approx 0.00051\left[\mathrm{rad}\right]$ for roll, $\mathrm{Max}\{\theta_{error}\}\approx 0.00079\left[\mathrm{rad}\right]$ for pitch, and $\mathrm{Max}\{\psi_{error}\}\approx 0.0013\left[\mathrm{rad}\right]$ for yaw (in Figure 9a), while the corresponding values by using the SMC are 0.0064 [rad] for roll, 0.0075 [rad] for pitch, and 0.0015 [rad] for yaw, and by using the ADRC are 0.033 [rad] for roll, 0.012 [rad] for pitch, and 0.0065 [rad] for yaw. In addition, the position control of UAV is also highly improved as exhibited in Figure 9b, the maximum tracking error of RBCESDO acquires about $\mathrm{Max}\{x_{error}\}\approx 0.009\left[m\right]$ for *x*, $\mathrm{Max}\{y_{error}\}\approx 0.0072\left[m\right]$ for *y*, $\mathrm{Max}\{z_{error}\}\approx 0.0002\left[m\right]$ for *z*, while the corresponding values by using the SMC are 0.22 [m] for *x*, 0.195 [m] for *y*, 0.0005 [m] for *z*, and by using the ADRC are 0.6 [m] for *x*, 0.22 [m] for *y*, 0.005 [m] for *z*. In summary, the proposed algorithm (RBCESDO) seems to provide a much better tracking performance for attitude and position control compared to the considered methods in both rapid response and minor tracking error.

## 5. Conclusions and Future Works

In this study, a robust backstepping control based on an extended state/disturbance observer for trajectory tracking is developed to improve the performance of the quadcopter UAV under the various influences of uncertainties and/or external perturbations. In the proposed algorithm, the ultimate control law is recursively constructed by using the ESDO method and defined auxiliary variables. The Lyapunov theorem is used to analyze and prove the robust stability and powerful convergence of the system states to reference paths. Finally, the numerical simulation is fully executed in both attitude and position control mode; these simulation results are also compared with the Sliding Mode Control and Active Disturbance Rejection Control methods to strongly confirm the superior performance of the presented control scheme in both desired trajectory convergence and anti-disturbance capacity. In addition, precise knowledge of the upper bounds of uncertainties and/or external disturbances is not demanded during the steps of designing the proposed controller.

Another significant contribution of the proposed algorithm comes from the real aspect. The ESDO is able to estimate both the velocity state of the UAV’s system and wind speed once these parameters cannot be directly measured due to sensor limitations. Thus, the real implementation cost for data acquisition may be reduced, and the influences of sensor noise are also removed. Future works are going to discover a finite-time convergence of the system states to a desired trajectory.

## Figures and Tables

**Table 1:** System parameters of a quadcopter UAV.

Symbol	Descriptions	Value and Unit
m	Total mass of the vehicle	1.12 kg
l	Arm length of quadcopter UAV frame	0.23 m
$J_{r}$	Inertial moment of a rotor	$8.5\;10^{-4}\;\mathrm{kg}.m^{2}$
$I_{x}$	Inertial moment around *x*-axis	$0.0019\;\mathrm{kg}.m^{2}$
$I_{y}$	Inertial moment around *y*-axis	$0.0019\;\mathrm{kg}.m^{2}$
$I_{z}$	Inertial moment around *z*-axis	$0.0223\;\mathrm{kg}.m^{2}$
b	Thrust coefficient	$7.73212\;\left(10^{-6}\right)\;N.s^{2}$
d	Drag coefficient	$1.27513\;\left(10^{-7}\right)\;N.m.s^{2}$

**Table 2:** Controller parameters for simulation.

Parameter	Descriptions	Value
α	Observer gain of matrix	diag[2.5, 2.5, 2,5, 5, 5, 5]
β	Observer gain of matrix	diag[100, 100, 100, 75, 75, 75]
$k_{1}$	Controller gain	diag[25, 25, 35, 12, 12, 8]
$k_{2}$	Controller gain	diag[18, 18, 35, 12, 12, 8]
$k_{3}$	Controller gain	diag[0.1, 0.1, 0.1, 0.1, 0.1, 0.1]
$Z_{0}$	Initial state value	[8, 0, 0, 0, 0, 0.1]

## Data Availability

Not applicable.
